# The skin is no barrier to mixtures: Air pollutant mixtures and reported psoriasis or eczema in the Personalized Environment and Genes Study (PEGS)

**DOI:** 10.1038/s41370-022-00502-0

**Published:** 2022-12-02

**Authors:** Melissa E. Lowe, Farida S. Akhtari, Taylor A. Potter, David C. Fargo, Charles P. Schmitt, Shepherd H. Schurman, Kristin M. Eccles, Alison Motsinger-Reif, Janet E. Hall, Kyle P. Messier

**Affiliations:** 1 National Institute of Environmental Health Sciences, Division of the National Toxicology Program, Durham, USA; 2 National Institute of Environmental Health Sciences, Clinical Research Branch, Durham, USA; 3 National Institute of Environmental Health Sciences, Biostatistics and Computational Biology Branch, Durham, USA; 4 National Institute of Environmental Health Sciences, Office of Data Science, Durham, USA; 5 National Institute on Aging, Clinical Research Core, Bethesda, USA; 6 National Institute on Minority Health and Health Disparities, Bethesda, USA

**Keywords:** Air pollution, Geospatial analyses, Dermal exposure, Criteria pollutants, Epidemiology

## Abstract

**Background:**

Autoimmune (AI) diseases appear to be a product of genetic predisposition and environmental triggers. Disruption of the skin barrier causes exacerbation of psoriasis/eczema. Oxidative stress is a mechanistic pathway for pathogenesis of the disease and is also a primary mechanism for the detrimental effects of air pollution.

**Methods:**

We evaluated the association between autoimmune skin diseases (psoriasis or eczema) and air pollutant mixtures in 9060 subjects from the Personalized Environment and Genes Study (PEGS) cohort. Pollutant exposure data on six criteria air pollutants are publicly available from the Center for Air, Climate, and Energy Solutions and the Atmospheric Composition Analysis Group. For increased spatial resolution, we included spatially cumulative exposure to volatile organic compounds from sites in the United States Environmental Protection Agency Toxic Release Inventory and the density of major roads within a 5 km radius of a participant’s address from the United States Geological Survey. We applied logistic regression with quantile g-computation, adjusting for age, sex, diagnosis with an autoimmune disease in family or self, and smoking history to evaluate the relationship between self-reported diagnosis of an AI skin condition and air pollution mixtures.

**Results:**

Only one air pollution variable, sulfate, was significant individually (OR = 1.06, *p* = 3.99E−2); however, the conditional odds ratio for the combined mixture components of PM_2.5_ (black carbon, sulfate, sea salt, and soil), CO, SO_2_, benzene, toluene, and ethylbenzene is 1.10 (*p*-value = 5.4E−3).

**Significance:**

While the etiology of autoimmune skin disorders is not clear, this study provides evidence that air pollutants are associated with an increased prevalence of these disorders. The results provide further evidence of potential health impacts of air pollution exposures on life-altering diseases.

**Significance and impact statement:**

- The impact of air pollution on non-pulmonary and cardiovascular diseases is understudied and under-reported. We find that air pollution significantly increased the odds of psoriasis or eczema in our cohort and the magnitude is comparable to the risk associated with smoking exposure. Autoimmune diseases like psoriasis and eczema are likely impacted by air pollution, particularly complex mixtures and our study underscores the importance of quantifying air pollution-associated risks in autoimmune disease.

## Introduction

Air pollution is recognized as a major contributor to excess mortality, primarily within cardiovascular and pulmonary diseases [1]. It is associated with increased hospitalizations and emergency room visits for COPD, asthma, and other chronic health problems [2]. The U.S. Environmental Protection Agency (EPA) monitors criteria air pollutants across the United States (US) with known detrimental health effects [3]. Some air pollutant mixtures such as those including particulate matter appear to have a high capacity for creating reactive oxygen species that initiate cellular inflammation and death [4].

Recent literature has suggested that environmental exposures may trigger autoimmune diseases [5]. Studies have also indicated an association between air pollution and skin diseases under the autoimmune umbrella [6–10]. Further, a recent retrospective cohort in South Korea found temporal associations between long-term exposure to a range of common air pollutants and the risk of developing psoriasis [11].

Atopic dermatitis (eczema) affects between 1–3% of adults and 15–20% of children [12]. It can diminish quality of life, alter social function, and cause substantial cost to patients, families, and health care systems. Similarly, psoriasis is estimated to affect up to 3.2% of the adult US population [13]. Psoriasis is a chronic skin disease that forms thick scaling plaques, causes itchy skin, irritation, pain, and soreness [14]. These diseases are a significant burden to patients and are associated with depression, anxiety, stress, stigmatization, and impaired quality of life in part due to limitations in disease management [15]. Insight into the potential pathogenesis and exacerbating triggers of these diseases is important for their prevention and management.

There is a strong genetic predisposition to both psoriasis and eczema. Atopic dermatitis and family history of psoriasis were significantly associated with pediatric psoriasis in a large pediatric cohort in Taiwan [7]. They also have morphologic characteristics that can be clinically difficult to distinguish [16]. Previous studies have shown that the oxidative stress mechanism associated with cardiovascular and lung diseases are likely shared with dermatological outcomes [4, 17].

We hypothesize that a proportion of autoimmune skin disease is associated with exposure to mixture air pollutants such as O_3_, SO_2_, NO_2_, coarse particles <0 µm in diameter (PM_10_), fine particles <2.5 µm in diameter (PM_2.5_), and BTEX chemicals(benzene, toluene, ethylbenzene, and xylene). Few studies have evaluated the association between pollutant mixtures and health outcomes although it is a growing area of research. Fewer still have explored autoimmune diseases in the context of air pollutant mixtures [18]. Researchers postulate that the health effects of air pollution are often underrepresented because single pollutant models fail to capture the complexity of the overall burden of multiple pollutants. To elucidate the joint effect of these pollutants on autoimmune skin disease, we applied quantile g-computation and adjusted for probable covariates [19]. We utilized home address data from over 9000 subjects within the Personalized Environment and Genes Study (PEGS) cohort to evaluate the association between average annual exposure to air pollutants and self-reported diagnosis of psoriasis or eczema.

## Methods

### Cohort description

The Personalized Environment and Genes Study (PEGS) is a diverse cohort with extensive health and exposure data at the National Institute of Environmental Health Sciences (NIEHS), (https://www.niehs.nih.gov/research/clinical/studies/pegs/index.cfm). Participants are mostly located in North Carolina (NC) with the remainder scattered across the contiguous United States.

The cohort began as a research registry in 2002 with recruitment from university campuses, health clinics and fairs, and volunteer study drives. From 2013 to 2020, subjects were administered the PEGS Health and Exposure Survey and two NIEHS PEGS Exposome Surveys (A and B).

The PEGS Health and Exposure Survey was formulated based on validated surveys like the National Health Information Survey and the National Health and Nutrition Examination Survey (NHANES) [20]. The Health and Exposure Survey and interactive tools to further inspect summary data can be found at https://www.niehs.nih.gov/research/clinical/studies/pegs/index.cfm.

The Health and Exposure Survey asks participants if they had a physician diagnosis of autoimmune diseases including multiple sclerosis, hyperthyroidism, hypothyroidism, Celiac, Crohns, ulcerative colitis, scleroderma, lupus, Sjogren’s syndrome, Raynaud’s phenomenon, pernicious anemia, myositis, rheumatoid arthritis, unspecified arthritis, psoriasis, and eczema. If subjects responded affirmatively, they were considered as having autoimmune disease.

Table 1 describes detailed characteristics of the PEGS cohort subjects.

**Table 1:** Characteristics of Cohort.

Characteristic	Structure	Value
*n*		9060
Age	mean (sd)	42.42 (15.84)
Gender (F)	count(%)	6082 (67.1)
**Race**	**count(%)**	
*American Indian/Alaska Native*		94 (1.0)
*Asian*		187 (2.1)
*Black*		1942 (21.4)
*Pacific Islander*		9 (0.1)
*White*		6504 (71.8)
*Multiple*		92 (10.1)
**Ethnicity**	**count(%)**	
*Hispanic/Latino*		347 (3.8)
Income (>$30,000)	count(%)	6654 (73.4)
Smoke >100 cigs	count(%)	3580 (39.5)
Autoimmune Disease (Y)	count(%)	3214 (35.5)
Psoriasis or Eczema	count(%)	1128 (12.5)
Family History of RA	count(%)	687 (7.6)

Our analysis included 9060 subjects with complete addresses. They were mostly middle-aged, 42 (15.8), with racial backgrounds reflecting the demographics of North Carolina residents [21]. The cohort is mostly female (67%) and relatively high-income. Almost 40% of subjects report smoking. Autoimmune diseases are relatively common with 35% reporting at least one of the above diseases. The prevalence of psoriasis and eczema is (4.2%) and (9.8%) respectively and together represent 1128 subjects (12.5%). Our outcome definition is a diagnosis of psoriasis and/or eczema.

### Air pollutant exposure estimation

We utilized two sources of pre-existing air pollutant data, one from the Center for Air, Climate, and Energy Solutions (CACES) https://www.caces.us/ and the other from the Atmospheric Composition Analysis Group (ACAG) http://fizz.phys.dal.ca/~atmos/martin/?page_id=140. The CACES estimates derive annual mean concentrations of the six criteria air pollutants by census tract in the contiguous United States from monitoring sites and approximately 350 geographic covariates in an integrated empirical geographic regression model [22]. Criteria air pollutant (NO_2_, SO_2_, O_3_, PM_2.5_, PM_10_, and CO) concentrations are monitored by the U.S. Environmental Protection Agency due to their known detrimental effects on health and are managed nationwide to comply with air quality standards. We linked the participant address data to the CACES data by selecting the nearest population-weighted census tract centroid to the participant address due to the format of the CACES data [23].

The ACAG estimated annual mean fine-level particulate matter (PM_2.5_) mass and compositional mass (total mass *µ*g/m^3^) across North America using Aerosol Optical Depth (AOD) estimates and the GEOS-Chem chemical transport model. PM_2.5_ composites include ammonia (NH_4_), sulfate (SO_4_), black carbon, nitrate, organic matter, soil, and sea salt. The estimates were calibrated to the ground-level using Geographically Weighted Regression (GWR) [24]. The ACAG values were matched to participant addresses using the raster R package’s extract function [25].

**Fig. 1:**
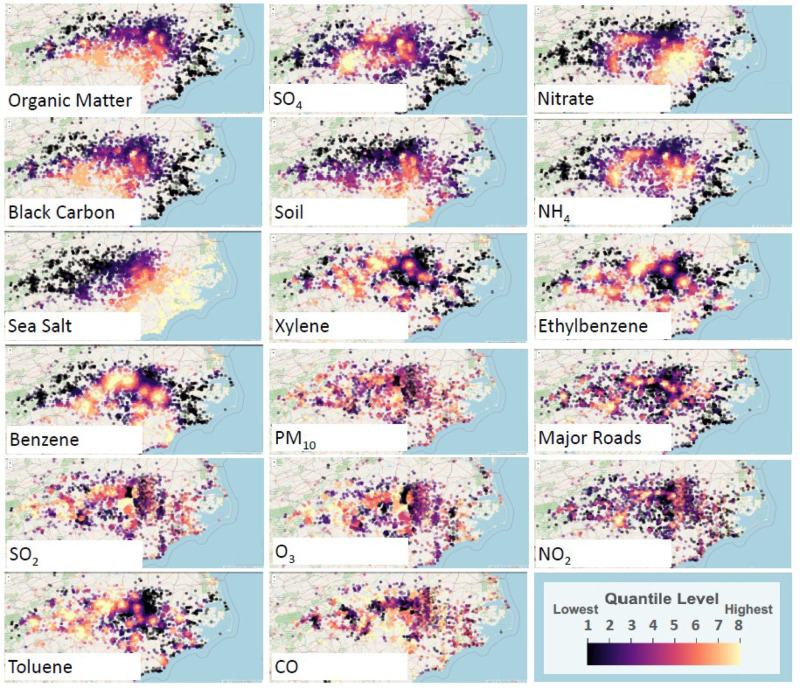
Maps of pollution intensity for each modeled constituent of the mixture. For comparability, the pollutants have been normalized, and eight quantiles were determined for each pollutant. Quantile 4 represents the mean exposure in the cohort. Each dot represents an approximate home address for subjects living in and around North Carolina although there are subjects living across the United States. Please see Table 2 for ranges of the original exposure values. Maps developed using leaflet package in R.

Since our subjects only had one address reported without information on the timeframe of residency and no temporal linkage to our outcome of interest, we chose to utilize the mean value across the years 2000–2015 for both the CACES data and for the ACAG data, see Fig. 1. Averaging over this period allowed for a reasonable estimates of typical air pollutant exposure following the establishment of the PEGS cohort registry. While both CACES and ACAG models utilize a wide-range of variable inputs, their output is generalized to a mid-range spatial resolution (census tract and 0.1^◦^lat/long grid). In order to increase the individual-level scale representation of pollutant exposures, we included two more exposure estimates – the density of major roads from the United States Geological

Survey (USGS) https://pubs.usgs.gov/dds/dds-059/export/metadata/akrds2mg.htm and the concentration of volatile organic compounds (VOCs) from the Toxic Release Inventory (TRI) https://www.epa.gov/toxics-release-inventory-tri-program. The VOCs included were benzene, ethylbenzene, toluene, and xylene (BTEX), commonly monitored constituents of petroleum products and representative of a diversity of sources of VOCs [26, 27]. The density of major roads was defined as the total length of roadways in a 5 km buffer around the participant address using the sf package in R [23]. We estimated for a 5 km buffer around the participant address the sum of the exponentially decayed mass (lb) from each TRI site based on the distance from the site to the participant’s address, see Eq. (1). The initial mass value is mean annual concentration at a site from 2000–2016.

1 $$X_i^k = \mathop {\sum}\limits_{i = 0}^{n_k} {C_{oj}{{{{{{{\mathrm{exp}}}}}}}}\left( { - 3\frac{{d_{ij}}}{{a_r}}} \right)}$$

where *X*_*i*_^*k*^ is variable *X* for location *i* and source type *k*, *C*_0*j*_ is the initial concentration at source *j* assumed to be the annual air release in kg as reported by the TRI, *d*_*ij*_ is the Euclidian distance between monitoring site *i* and source *j*, *a*_*r*_ is the exponential decay range, and *n*_*k*_ is the number of source of type *k* [28].

Mean, standard deviations, and ranges for the exposures included can be found in Table 2, while comparative quantiles of pollutants are mapped in Fig. 1.

**Table 2:** Exposure quantity ranges in cohort with units specified.

Exposure	Mean (SD)	Range	Unit
NH_4_, PM_2.5_	1.050 (0.160)	(0.147, 2.13)	Micrograms/m^{3}
Black Carbon, PM_2.5_	0.785 (0.120)	(0.212, 3.023)	Micrograms/m^{3}
Nitrate, PM_2.5_	0.722 (0.297)	(0.041, 5.459)	Micrograms/m^{3}
Organic Matter, PM_2.5_	3.437 (0.522)	(1.000, 10.182)	Micrograms/m^{3}
SO4, PM_2.5_	3.154 (0.390)	(0.323, 4.459)	Micrograms/m^{3}
Sea Salt, PM_2.5_	0.286 (0.150)	(0.012, 2.723)	Micrograms/m^{3}
Soil, PM_2.5_	0.442 (0.116)	(0.118, 2.894)	Micrograms/m^{3}
CO	0.343 (0.059)	(0.208, 0.757)	ppm
NO_2_	6.891 (2.664)	(1.394, 29.393)	ppb
O_3_	42.691 (7.719)	(29.480, 60.012)	ppb
PM_10_	15.265 (4.416)	(5.444, 44.959)	Micrograms/m^{3}
SO_2_	1.874 (0.785)	(0.708, 7.067)	ppb
Benzene	19.820 (235.69)	(0, 12643.11)	Pounds per 5 km radius
Ethylbenzene	33.49 (372.06)	(0, 14947.96)	Pounds per 5 km radius
Toluene	839.47 (5268.06)	(0, 181626.3)	Pounds per 5 km radius
Xylene	356.90 (1715.24)	(0, 47517.2)	Pounds per 5 km radius
Road density	1444.89 (1505.21)	(0, 10530.76)	Km

We note in Fig. 1 that many pollutants such as the BTEX chemicals, SO_4_, and NO_2_ cluster around the urban corridors in North Carolina. Of further interest, nitrate and ammonia (*NH*_4_) constituents of PM_2.5_ have spatial patterns following the density of confined area feeding operations where animal waste is highly concentrated [29]. We also note a wide range of exposures for home locations in the cohort.

### Covariates

We used a directed acyclic graph (DAG) to show covariates in relationships with an approach modeled after a causal inference framework; however, there are assumptions that our dataset does not meet for inference of causal estimates [30]. Nonetheless, the approach allows explicit representation of perceived factors modifying our outcome.

**Fig. 2:**
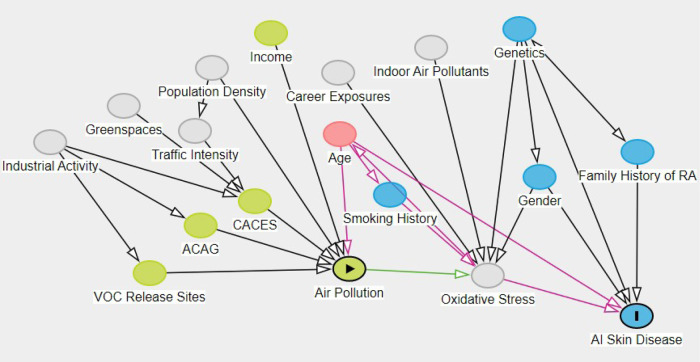
Directed acyclic graph showing hypothesized relationships between air pollution, our included covariates, and our outcome of autoimmune skin diseases. The green circle with arrow-head represent our primary mixture exposure; the green circles represent the air pollution exposures; grey circles represent air pollution sources or mitigation factors; the blue circle with an I is our outcome of interest, and the plain blue circles are biological covariates. Pink represents confounders and shows that the minimally sufficient set of covariates is the subject’s age.

The covariates that we included in the model describe potential pathways of exposure that influence our outcome. Additionally, there are likely unmeasured confounders that impact known exposures included in the model. We included the postulated relationships of exposures and other covariates to the outcome in the DAG [31], see Fig. 2.

The selected exposures describe the influence of population density that drives traffic intensity, industrial activity that drives the production of VOCs with both driving the quantity of criteria air pollutants. CACES in its estimation also includes greenspaces which have been shown to modify the intensity of air pollutants in neighborhoods [24].

Socioeconomic status is an important determinant of home location which is used as a proxy for pollutant exposure. Socioeconomic status is also co-linked with career and therefore career-related exposures. While the PEGS cohort does have substantial data gathered on career-related exposures, they are numerous and poorly defined for the scope of this analysis. For sensitivity analysis, we included a dichotomous variable indicating whether subjects had incomes of more than $30,000. This cutoff approximated living wage calculations in NC as of 2019 [32], see table S2 and https://livingwage.mit.edu/pages/about and poverty levels across the U.S. for households of 4–5 people [21] (https://www.census.gov/data/tables/time-series/demo/income-poverty/historical-poverty-thresholds.html).

Autoimmune diseases have strong genetic linkages and to reflect this, we included whether subjects had one or more parent with rheumatoid arthritis or if a subject reported other autoimmune conditions, inclusion was also considered in sensitivity analyses, see Table S1. Age and gender were included to adjust for the sample distribution. Age is also a confounder as it is associated with the amount of overall exposure to the pollutant mixtures and the probability of diagnosis and is considered the extent of the minimally sufficient set of covariates to elucidate the pathway of interest.

Smoking status and history are associated with autoimmune disease and severity as well as most health conditions. Smoking exposure is also a major contributor to oxidative stress and therefore was adjusted for in this analysis [33]. PEGS subjects were asked about current smoking and smoking history. We included an indicator of whether subjects had reported smoking more than 100 cigarettes in their lifetime to adjust primarily for habitual smokers.

### Statistical analysis

To evaluate the joint association of a mixture of air pollutants with autoimmune skin disease, we utilized quantile g-computation as described by Keil et al. 2020 [19]. The outcome, self-reported diagnosis of psoriasis and/or eczema is a binary time-fixed variable, hence we estimated mixture effect within a logistic regression structure.

G-computation approximates potential counterfactual situations by estimating the outcome in the circumstance for all possible values of exposure through resampling the dataset. Under the assumptions of no unmeasured confounders and exchangeability, g-computation fits the actual data to a model and then predicts outcomes for potential counterfactual data [34]. Once the counterfactual dataset is created, a marginal structural model is estimated by regressing with the data including the counterfactuals [34]. Therefore, the model based on collected data is adjusted for data-estimated counterfactual situations. Quantile g-computation is a mixtures-motivated extension of g-computation that estimates the effect of simultaneously increasing each pollutant by one quantile (*q*) for a subject *i* of exposure *j*. In Eq. (2), $X_{i,j}^q$ represent the geospatially-linked quantity of air pollutants estimated at subject *i*’s home address and *Z*_*ik*_ represent the *k* other covariates including gender, age, smoking history, and family history of autoimmune conditions (one or more parent with rheumatoid arthritis). *α*_*k*_ being the coefficient estimates for the non-exposure covariates.

2 $$Logit\left( {P\left( {Y_i{{{{{{{\mathrm{|}}}}}}}}X^q} \right)} \right) = \beta _o + \mathop {\sum}\limits_{j = 1}^d {\beta _jX_{ij}^q + \alpha _kZ_{ik} + \varepsilon _i}$$

The final regression estimates the marginal effect of the mixture, *ψ*, for a quantile increase in the combined exposure variables where $\psi = \mathop {\sum}\nolimits_{j = 1}^d {\beta _j}$ [19]. To establish consistent and comparable quantiles, all exposures were first scaled to a zero mean and then divided into 8 quantiles. Octiles are a comprehensible unit and allow for adequate variability to be expressed between quantiles without losing substantial information. The test statistics and confidence intervals were then derived from 1000 bootstrap samples. All models were run using the R package, qgcomp 2.8.6 [35].

## Results

The quantile g-computation method suggests that correlated exposures (Fig. S3) hypothesized to be causal should be included in the estimate of *ψ* to avoid any confounding of co-pollutants [19]. We thus included all measured pollutants in the estimate of *ψ*. Income was originally included in the model but had an insignificant and small effect size and was removed from the final model, see Table S2.

Table 3 presents the risk ratios, confidence intervals, *p*-values, and joint effect weights from our analysis while Fig. S2 visually represents the weights of exposures in *ψ*. The mixture value of *ψ* was found to be significant and to increase the risk of diagnosis by 10%, (CI: 1.03,1.17). Within the mixture constituents, we see that sulfate (SO_4_) increases the probability of autoimmune skin disease by 6% and represents the only constituent that has an independent and significant association with the outcome. We tested the estimate of *ψ* without SO_4_ and found that the estimate of *ψ* decreased by 0.02, see Table S3. SO_4_, and NH_4_ dominate the relative weights in *ψ* suggesting that their contribution to the effect is substantial.

**Table 3:** Results from logistic regression using Quantile G-Computation Results with 1000 bootstraps.

Coefficient	Odds Ratio	Conf. Int.	*P*-Value	Weight
Intercept	0.115	(0.082,0.161)	<2.2E−16*	
100+ Cigarettes	1.164	(1.022, 1.325)	2.20E−2*	
Age	0.992	(0.988, 0.996)	1.90E−4*	
Gender (M)	0.812	(0.705, 0.935)	3.79E−3*	
Family History	1.210	(0.971, 1.51)	9.02E−2	
Any other AI Disease	1.678	(1.46, 1.931)	5.30E−13*	
**PSI**	**1.1**	**(1.028, 1.174)**	**5.39E**−3*	
NH_{4}, PM_{2.5}	0.976	(0.910, 1.050)	5.36E−1	−0.3021
Black Carbon, PM_{2.5}	1.004	(0.928, 1.093)	8.66E−1	0.0195
Nitrate, PM_{2.5}	0.988	(0.935, 1.044)	6.63E−1	−0.1437
Organic Matter, PM_{2.5}	0.988	(0.918, 1.067)	7.90E−1	−0.1475
SO_{4}, PM_{2.5}	1.059	(1.002 1.115)	3.99E−2*	0.3028
Sea Salt, PM_{2.5}	1.023	(0.990, 1.065)	1.58E−1	0.1226
Soil, PM_{2.5}	1.027	(0.974, 1.066)	4.04E−1	0.1406
CO	1.005	(0.975, 1.036)	7.44E−1	0.0276
NO_{2}	0.995	(0.963,1.030)	8.12E−1	−0.0560
O_{3}	0.995	(0.953, 1.039)	8.12E−1	−0.0660
PM_{10}	0.999	(0.952, 1.029)	6.01e−1	−0.1322
SO_{2}	1.019	(0.974,1.066)	4.25E−1	0.0992
Benzene	1.019	(0.986,1.054)	2.52E−1	0.1027
Ethylbenzene	1.025	(0.983,1.068)	2.50E−1	0.1320
Toluene	1.003	(0.961,1.045)	9.15E−01	0.0143
Xylene	0.988	(0.943,1.037)	6.42E−1	−0.1525
Road Density	1.007	(0.974,1.041)	6.74E−1	0.0385

A risk ratio >1 indicates the component elevates the probability of psoriasis or eczema. Significant risk ratios, *p* ≤ 0.05 are marked with *. We also note the relative weights of the mixture constituents for the joint effect of *ψ*.

Family history of rheumatoid arthritis and the presence of other autoimmune diseases had the strongest association with an increased probability of psoriasis diagnosis with odds ratios of 1.21 and 1.68, respectively, although the family history did not cross the significance threshold, see Table S1. Male subjects had a lower probability of diagnosis (OR = 0.812) and each additional year of age at entry to the cohort lowered the probability of diagnosis by a small but significant amount (OR = 0.992). Smoking history also increased the probability of diagnosis by a significant and clinically relevant amount of 16%.

## Discussion

This study uniquely evaluates the impact of a complex mixture of air pollutant exposures on a rarely studied outcome of autoimmune skin diseases in association with large-scale environmental exposures. Our results indicate a positive association between a cumulative burden of exposure to multiple air pollutants and the reported diagnosis of psoriasis and/or eczema. Furthermore, we found an association between air pollution exposure and disease that may not be seen in single-pollutant models. The multi-pollutant effect remains following adjustment for covariates including the presence of other autoimmune conditions, a family history of an autoimmune disease (rheumatoid arthritis), and a moderate history of smoking (> 100 cigarettes).

The exposome was first suggested as an epistemological approach to characterize the environmental drivers of disease [36]. One of the key issues in addressing the impact of the exposome on health is modeling of the complex mix of exposures that humans experience. Air pollution exposure is not singularly sourced and the multiple pollutants and sources undoubtedly have synergistic interactions and effects [37]. For realistic policy interventions and air pollution criteria, the health impact of pollutant mixtures needs to be quantified [37]. In this analysis, we find a strong indication of health impact from multi-pollutant exposure on skin disease where an additional octile of air pollution burden increases the probability of autoimmune skin disease by 10% that is not identified if pollutants are covariates in a simple logistic regression, see Table S4.

Among the mixture constituents, an increase in the sulfate (SO_4_) proportion of PM_2.5_ increased the probability of autoimmune skin disease by 6% (CI:1.003, 1.115) and was the only constituent with an independent, significant association with the outcome. Atmospheric sulfate is the result of primary pollutant emissions and atmospheric reactions from SO_2_ related to anthropogenic and natural sources [38]. Sulfate increases metal solubility and oxidant formation through acid dissolution [38]. The combination of small particle size (geometric mean diameter = 0.97 *µ*m of sulfate particles [38]), high oxidative potential and its relatively large proportion of PM_2.5_ mass suggests that sulfate may represent a large portion of the particles capable of entering the lungs and potentially crossing the air-blood barrier [24, 38, 39].

We again note that within this study, there was either inadequate power for detection or few of the constituents had an effect large enough to have a significant impact on the probability of diagnosis independently of the other pollutants in the mixture. However, the probability of disease associated with a one-octile increase in pollutant exposure across the whole mixture (OR = 1.10, (1.03,1.17)) is comparable to having a substantial history of smoking (OR = 1.16, (1.02,1.32)). This strength of association supports a postulated causal relationship between exposure to air pollution and the prevalence of life-disrupting autoimmune diseases.

Family history of autoimmune disease and the diagnosis of other autoimmune diseases are strongly associated with psoriasis or eczema diagnoses. This result is supported by previous findings that autoimmune diseases often occur concurrently [40]. The current cohort questionnaire has a limited family medical history and the non-significance of the family history of rheumatoid arthritis may be related to other unknown familial traits such that the effect might be strengthened through gene-by-environment analysis. The hypothesized adverse outcome pathway of increased reactive oxygen species (ROS) production affecting atopic dermatitis and psoriasis likely impacts other autoimmune diseases similarly, again motivating the use of gene-by-environment methods [41].

Male subjects had a lower probability of diagnosis which is consistent with observations that the majority of autoimmune disease patients are female [42]. This may be due to sex differences in the production of reactive oxygen species and biologic susceptibility to oxidative stress [43]. A moderate smoking history (>100 cigarettes) was associated with an increased probability of diagnosis. This association is unsurprising as smoking is known to exacerbate and cause a wide range of diseases including autoimmune conditions [40].

We applied a sensitivity analysis including an indicator variable for income less than or equal to $30,000 annually. Income was not a significant predictor of psoriasis or eczema diagnosis in this cohort. We hypothesize, however, that income is strongly correlated with home location and home location defines the estimate of exposure. This is especially true for the association of high traffic density areas which are spatially associated with higher combustion-related exposures and lower incomes, see correlation of urban areas with pollutants in Fig. 1 [44].

Previous research relating air pollution exposure to psoriasis or eczema has been done in multiple countries. Ecological epidemiology studies including outpatient visits in Turkey and emergency department visits in Canada saw increases in particulate matter, SO_2_, O_3_, and NO_2_ temporally associated with increased healthcare utilization [6, 45]. In Japan, policy interventions begun in 2001 to manage automobile NO_*x*_ and particulate matter resulted in decreased incidence of asthma in areas with successful intervention and increased incidence of atopic dermatitis in areas without intervention where ambient pollution increased [8]. A 2017 meta-analysis of human skin diseases and particulate matter exposure found consistent associations between atopic dermatitis and PM_2.5_ exposure [9].

Beyond the population-level associations suggested by previous studies, there is biologic evidence that air pollution has detrimental impacts on patients with atopic dermatitis, eczema, and psoriasis [46–48]. Several physiological mechanisms have been proposed to explain the impact of air pollutant exposure on human health. One important area is the role of air pollution in driving the creation of ROS and reactive nitrogen species (RNS) in human tissue. Specifically, ROS and RNS have been reported to modify proteins and increase lipid peroxidation in autoimmune diseases in patients and in mouse models by initiating oxidative and nitrosative stress [5, 47, 49].

Certain biomarkers such as urinary 8-hydroxyl-2-deoxyguanosin, associated with oxidative stress in atopic dermatitis patients were found to increase in the 24 hours following exposure to ultrafine particles and polycyclic aromatic hydrocarbons [46]. Few studies have evaluated the association of psoriatic lesions with air pollution; however, a recent study found that ultrafine particles cause up-regulation of inflammatory and psoriasis-related genes and disrupted differentiation of keratinocytes in an in-vitro model [50]. In general, patients with atopic dermatitis and psoriasis tend to have higher levels of free radical generation, peroxidation resistance, and activity of antioxidant/antiradical defense when cells and biomarkers were studied [10, 12].

Thus, air pollutants have been temporally linked to changes in eczema and psoriasis symptoms and biomarkers as well as identified through in-vitro studies as being biologically plausible initiators of skin-barrier disruption and oxidative stress. There is increasing awareness that human disease is the result of complex interactions between the exposome and genome. Many studies also show correlations between common air pollutants vary dependent on spatial and temporal associations [51]. We uniquely explore the impact of a broad section of the exposome on these skin diseases by applying quantile g-computation to air pollutant mixtures.

This analysis affirms results from previous studies and further suggests that the risk of autoimmune skin disease is heightened by exposure to a complex mixture of pollutants that previously had been singularly associated with poor outcomes in psoriasis and eczema [6, 8, 9, 12, 17, 45, 46, 50, 52]. We identify the joint effect of pollutant mixtures and further find that sulfate exposure may be a primary contributor to autoimmune skin disease manifestation. While PEGS was not designed to study causal relationships, we utilized quantile g-computation to create a reasonable and robust structure for a counterfactual of exposure. We also recognize that atmospheric chemicals have complex and non-linear relationships with each other that were not tested in this model. However, the organizational methodology behind the direct acyclic graphs, the standardization of co-pollutants, and marginal structural model adjusted for counterfactuals all help to minimize bias and the influence of unmeasured confounders.

We note that this study evaluated the association of ambient air pollution with autoimmune skin disease. The indoor environment is also likely to influence the development and exacerbation of skin diseases [53, 54]. The PEGS cohort did collect survey data on indoor environmental and career exposures but it was not available for analysis at the time of this publication.

One of the strengths of our study is the sample size of more than 9000 subjects representative of the ethnic and racial diversity present in North Carolina. Home location as a proxy is common in studies of air pollution as long-term monitoring of air pollutants at an individual-level is cost-prohibitive and technologically difficult. We note that the time of diagnosis and time of exposure are unspecified in our data, however, our exposure estimate was conservative as a 15-year mean of annual concentration is likely to underestimate overall individual exposure given that emissions have been reduced since 1990 [55], see Supplemental Figs. S3, S4.

It should be noted that many of the subjects in the PEGS cohort live in areas that meet the EPA recommendations for criteria air pollutant levels. No counties in North Carolina are currently on the EPA Nonattainment Counties List https://www3.epa.gov/airquality/greenbook/ancl.html. We suggest that the risk ratios found in this analysis underestimate the increased risk of autoimmune skin disease that may occur in populations experiencing greater air pollution burdens especially in urban and industrial centers. It also gives further weight to the argument that attainment goals should be set to more aggressive levels of pollutant management with the recognition that there are detrimental health impacts at lower levels of air pollution than what is currently expected [56].

In future work, we will evaluate a potential gene-by-environment interactions for autoimmune skin diseases and air pollutants. Autoimmune diseases have a strong genetic component and the adjustment for parental rheumatoid arthritis suggests that the increase in the odds of disease development in relation to a gene-environment interaction may be significant.

## Supplementary information

Reporting Checklist

Supplemental Document

## Data Availability

The datasets generated during and/or analyzed during the current study are available from the corresponding author on reasonable request.
