# Supplementary material for: The skin is no barrier to mixtures: Air pollutant mixtures and reported psoriasis or eczema in the Personalized Environment and Genes Study (PEGS)
Source: J Expo Sci Environ Epidemiol. 2022 Dec 2;33(3):474–81. doi: 10.1038/s41370-022-00502-0 (PMC10234803; doi:10.1038/s41370-022-00502-0)

S1 Supplemental Document

The following tables and figures are supplemental materials for the manuscript, The skin is no barrier to mixtures: Air pollutant mixtures and reported psoriasis or eczema in the Personalized Environment and Genes Study (PEGS).

Figure S1: This figure shows the relationship between the correlation of two exposures and the confidence interval width around ψ (Psi) in blue and β_1, exposure 1's coefficient in red after adjustment for the other non-exposure confounders in the model. We tested the relationship between the correlation of a random selection of exposures in the dataset and the associated confidence interval width around ψ and found that the confidence interval width decreased in association with higher correlation between exposures indicating that the quantile g-computation estimator of ψ was performing correctly.

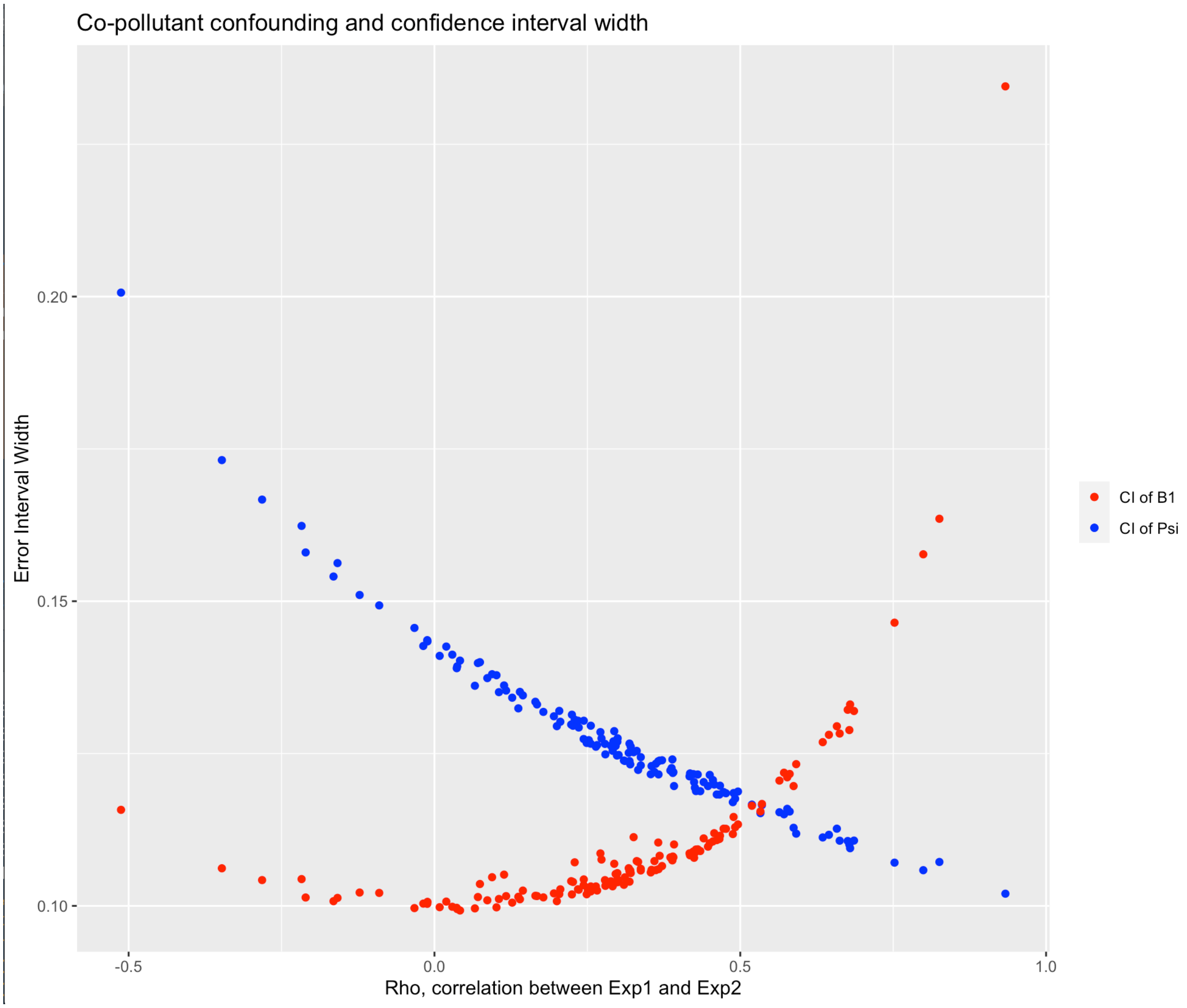

Figure S2: This figure shows the relative weights of contribution to the joint effect estimate of $\boldsymbol{\psi}$ as estimated by the quantile g-computation method. Here, we can see that SO_4 and NH_4 dominate the joint effects with NH_4 protective and SO_4 detrimental

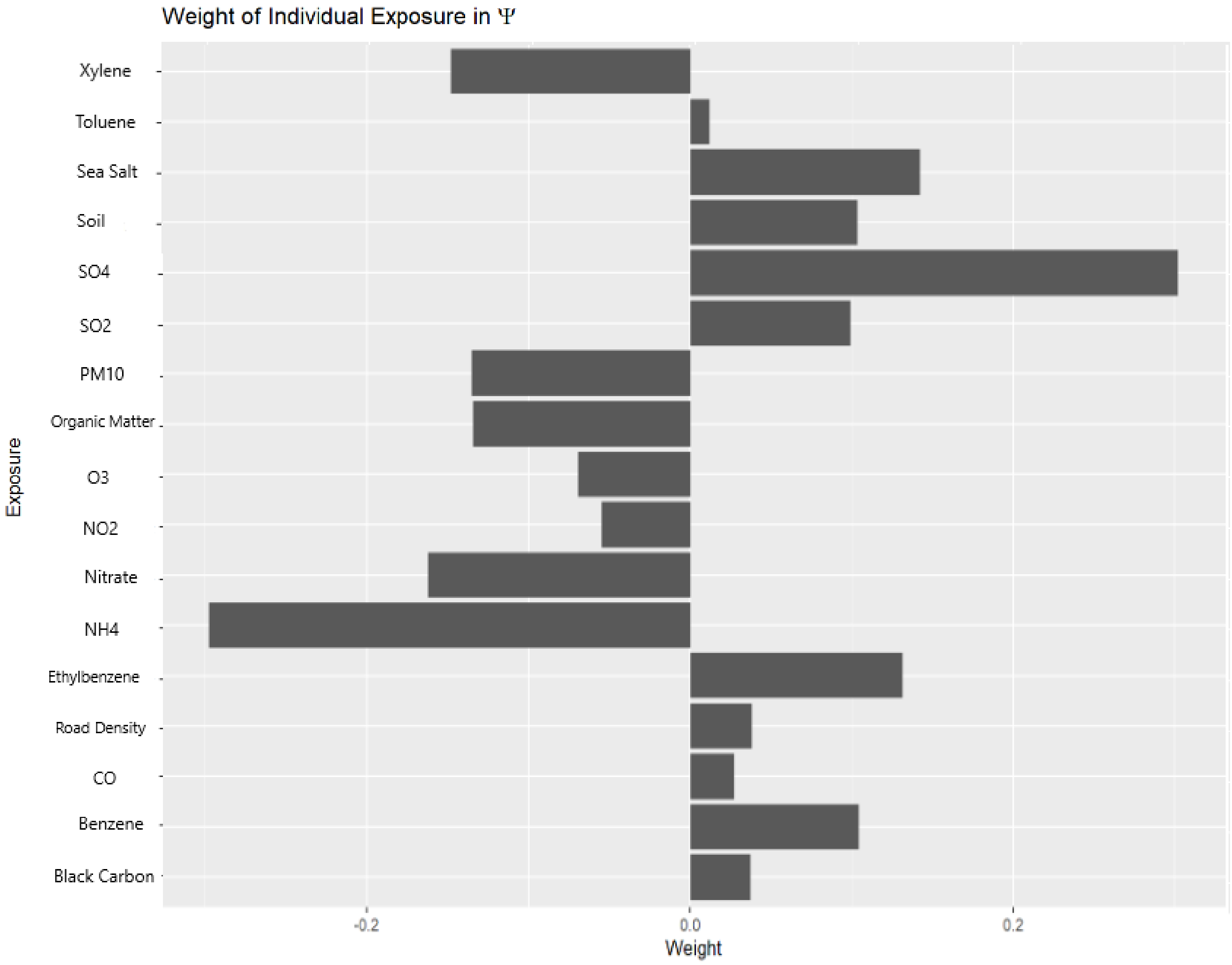

Figure S3: This figure shows the correlations between the proposed exposures included in the estimate of the joint effect.

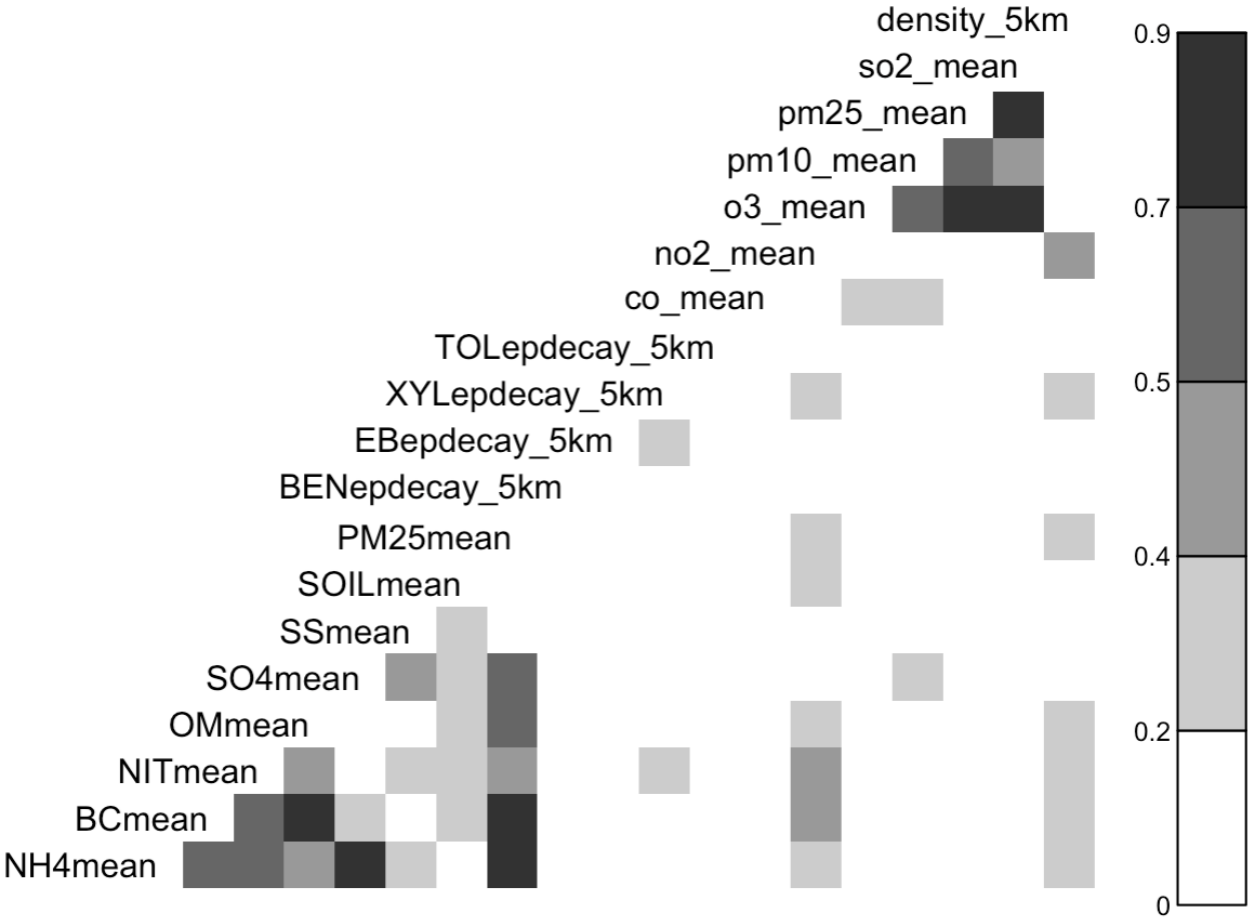

Table S1: Effect of removing parental rheumatoid arthritis diagnosis on logistic regression using Quantile G-Computation, results with 1000 bootstraps. An odds ratio > 1 indicates the component elevates the probability of psoriasis or eczema. Significant risk ratios, $p \leq 0.05$ are marked with *. Here, we note little change to covariate effect estimates. We note that familial history of RA is a weak indicator of genetic history but has a reasonable biologic postulate behind its inclusion.

**Coefficient**	**Odds Ratio**	**(Conf. Interval)**	**P-Value**
**Intercept**	0.116	(0.083,0.163)	2.76E-36 *
**100+ Cigarettes**	1.166	(1.023,1.327)	2.08E-2*
**Age**	0.992	(0.988.0.996)	2.40E-4*
**Gender (M)**	0.808	(0.702,0.931)	3.104633E-3*
**Any other AI Disease**	1.694	(1.473, 1.949)	1.492E-13*
$\boldsymbol{\psi}$	**1.097**	(1.028, 1.173)	**0.006***
**NH_4, $PM_{2.5}$**	0.975	(0.909,1.049)	5.16E-1
**Black Carbon, $PM_{2.5}$**	1.004	(0.929,1.093)	8.55E-1
**Nitrate, $PM_{2.5}$**	0.989	(0.936,1.045)	6.89E-1
**Organic Matter, $PM_{2.5}$**	0.988	(0.917,1.067)	7.81E-1
**SO_4, $PM_{2.5}$**	1.057	(1.003, 1.11)	3.92E-2*
**Sea Salt, $PM_{2.5}$**	1.023	(0.989,1.065)	1.66E-1
**Soil, $PM_{2.5}$**	1.027	(0.975,1.067)	3.95E-1
**CO**	1.005	(0.975,1.036)	7.49E-1
**NO_2**	0.996	(0.963,1.030)	8.18E-1
**O_3**	0.995	(0.953,1.039)	8.16E-1
**PM_{10}**	0.990	(0.952,1.029)	6.03E-1
**SO_2**	1.019	(0.973, 1.065)	4.25E-1
**Benzene**	1.019	(0.986, 1.053)	2.65E-1
**Ethylbenzene**	1.025	(0.983, 1.068)	2.50E-1
**Toluene**	1.003	(0.962, 1.045)	9.05E-1
**Xylene**	0.987	(0.943,1.036)	6.26E-1
**Road Density**	1.007	(0.974,1.041)	6.95E-1

Table S2: Effect of including indicator variable for annual incomes $\leq \$30,000$ om logistic regression using Quantile G-Computation, results with 1000 bootstraps. A risk ratio > 1 indicates the component elevates the probability of psoriasis or eczema. Significant risk ratios, p $\leq$ 0.05 are marked with *. Here, we include income as a covariate creating an indicator variable for subjects under the living wage approximation in NC. However, not all subjects responded to the income questionnaire and family size dependent on the income was not recorded. We note that income is not significant and did not change the effect size of psi or any covariates in a significant way.

Coefficient	Odds Ratio	Conf. Interval	P-Value
**Intercept**	0.108	(0.075,0.154)	5.62E-34*
**100+ Cigarettes**	1.174	(1.030,1.338)	1.64E-2*
**Age**	0.992	(0.988,0.996)	1.65E-4*
**Gender (M)**	0.809	(0.702,0.932)	3.23E-3*
**Family History**	1.214	(0.974, 1.514)	8.52E-2
**Income** $\leq \$30,000$	1.080	(0.927, 1.259)	3.24E-1
**Any other AI Disease**	1.683	(1.462,1.937)	3.94E-13*
$\boldsymbol{\psi}$	**1.100**	(1.030, 1.176)	**4.75E-3***
**NH_4, $PM_{2.5}$**	0.978	(0.911, 1.051)	5.47E-1
**Black Carbon, $PM_{2.5}$**	1.007	(0.928,1.092)	8.75E-1
**Nitrate, $PM_{2.5}$**	0.988	(0.935,1.044)	6.62E-1
**Organic Matter, $PM_{2.5}$**	0.991	(0.919,1.068)	8.09E-1
**SO_4, $PM_{2.5}$**	1.057	(1.002, 1.114)	4.27E-2*
**Sea Salt, $PM_{2.5}$**	1.027	(0.990,1.066)	1.47E-1
**Soil, $PM_{2.5}$**	1.019	(0.974,1.066)	4.13E-1
**CO**	1.005	(0.975, 1.036)	7.49E-1
**NO_2**	0.996	(0.963, 1.030)	8.18E-1
**O_3**	0.995	(0.953, 1.039)	8.13E-1
**PM_{10}**	0.989	(0.952, 1.028)	5.82E-1
**SO_2**	1.019	(0.974,1.066)	4.10E-1
**Benzene**	1.018	(0.985,1.053)	2.77E-1
**Ethylbenzene**	1.024	(0.983, 1.068)	2.57E-1
**Toluene**	1.003	(0.962, 1.046)	8.81E-1
**Xylene**	0.988	(0.943,1.036)	6.30E-1
**Road Density**	1.010	(0.976,1.043)	6.07E-1

Table S3: Effect of removing SO_4 from $\boldsymbol{\psi}$ and model estimation for logistic regression using Quantile G-Computation, results with 1000 bootstraps. A risk ratio > 1 indicates the component elevates the probability of psoriasis or eczema. Significant risk ratios, $p \leq 0.05$ are marked with *. We note that no other individual $\boldsymbol{\psi}$ became significant while the overall value of $\boldsymbol{\psi}$ decreased but maintained a significant effect size.

Coefficient	Odds Ratio	Conf. Interval	P-Value
**Intercept**	0.123	(0.088 ,0.170)	6.63E-36 *
**100+ Cigarettes**	1.161	(1.020 ,1.322)	2.39E-2*
**Age**	0.992	(0.988 ,0.996)	2.823E-4*
**Gender (M)**	0.811	(0.704, 0.934)	3.68E-3*
**Family History**	1.212	(0.972, 1.511)	8.79E-2
**Any other AI Disease**	1.674	(1.455 ,1.926)	6.49E-13*
$\boldsymbol{\psi}$	**1.08**	(1.011,1.153)	**0.02***
**NH_4, $PM_{2.5}$**	1.026	(0.971, 1.084)	3.54E-1
**Black Carbon, $PM_{2.5}$**	1.021	(0.943, 1.106)	6.07E-1
**Nitrate, $PM_{2.5}$**	0.962	(0.916, 1.011)	1.29E-1
**Organic Matter, $PM_{2.5}$**	0.993	(0.921, 1.070)	8.53E-1
**Sea Salt, $PM_{2.5}$**	1.023	(0.987, 1.061)	2.17E-1
**Soil, $PM_{2.5}$**	1.016	(0.972 ,1.063)	4.79E-1
**CO**	1.005	(0.975, 1.036)	7.57E-1
**NO_2**	0.998	(0.965, 1.033)	9.12E-1
**O_3**	0.990	(0.949, 1.033)	6.48E-1
**PM_{10}**	0.985	(0.948, 1.024)	4.54E-1
**SO_2**	1.023	(0.978, 1.070)	3.17E-1
**Benzene**	1.023	(0.990 ,1.057)	1.79E-1
**Ethylbenzene**	1.024	(0.982, 1.067)	2.64E-1
**Toluene**	0.997	(0.956, 1.039)	8.72E-1
**Xylene**	0.996	(0.950, 1.043)	8.55E-1
**Road Density**	1.009	(0.976 ,1.043)	5.97E-1

Table S4: Modeling association of air pollution on psoriasis/eczema without the mixture structure associated with quantile g-compution. Here, we use logistic regression with pollutants structured as covariates only. There is no estimate of a group effect. A ratio > 1 indicates the variable elevates the probability of psoriasis or eczema. Significant ratios, $p \leq 0.05$ are marked with *. We note that the individual pollutants do not have significant effects without the additional adjustment for their mixture-based contribution except xylene which is not significant in the gcomputation model nor a great contributor to the relative weights of $\boldsymbol{\psi}$.

Coefficient	Odds Ratio	Conf. Interval	P-Value
**Intercept**	0.1675	(0.139 ,0.202)	6.75E-79*
**100+ Cigarettes**	1.150	(1.010, 1.309)	3.438351E-2*
**Age**	0.992	(0.988, 0.996)	3.047E-4*
**Gender (M)**	0.810	(0.703, 0.9326686)	3.42E-3*
**Family History**	1.208	(0.968 ,1.507)	9.37E-2*
**Any other AI Disease**	1.673	(1.454, 1.926)	6.69E-13*
**NH_4, $PM_{2.5}$**	0.979	(0.688 ,1.393)	9.08E-1
**Black Carbon, $PM_{2.5}$**	0.890	(0.729, 1.086)	2.51E-1
**Nitrate, $PM_{2.5}$**	1.039	(0.799, 1.351)	7.726E-1
**Organic Matter, $PM_{2.5}$**	1.104	(0.937, 1.301)	2.37E-1
**SO_4, $PM_{2.5}$**	1.089	(0.819, 1.448)	5.57E-1
**Sea Salt, $PM_{2.5}$**	1.030	(0.948, 1.118)	4.83E-1
**Soil, $PM_{2.5}$**	1.034	(0.959, 1.114)	3.87E-1
**CO**	0.996	(0.919, 1.081)	9.34E-1
**NO_2**	1.009	(0.933, 1.091)	8.24E-1
**O_3**	0.985	(0.876, 1.108)	8.03E-1
**PM_{10}**	0.976	(0.884, 1.077)	6.27E-1
**SO_2**	1.037	(0.939, 1.145)	4.73E-1
**Benzene**	1.005	(0.950 ,1.064)	8.52E-1
**Ethylbenzene**	1.027	(0.975, 1.082)	3.101E-1
**Toluene**	0.963	(0.890, 1.043)	3.58E-1
**Xylene**	1.067	(1.011 ,1.126)	1.84E-2*
**Road Density**	1.030	(0.958, 1.109)	4.22E-1

Figure S4: This figure shows the annual average for criteria air pollutants and $PM_{2.5}$ components. In general, we see that the mean values have decreased across the 15+ years of collection.

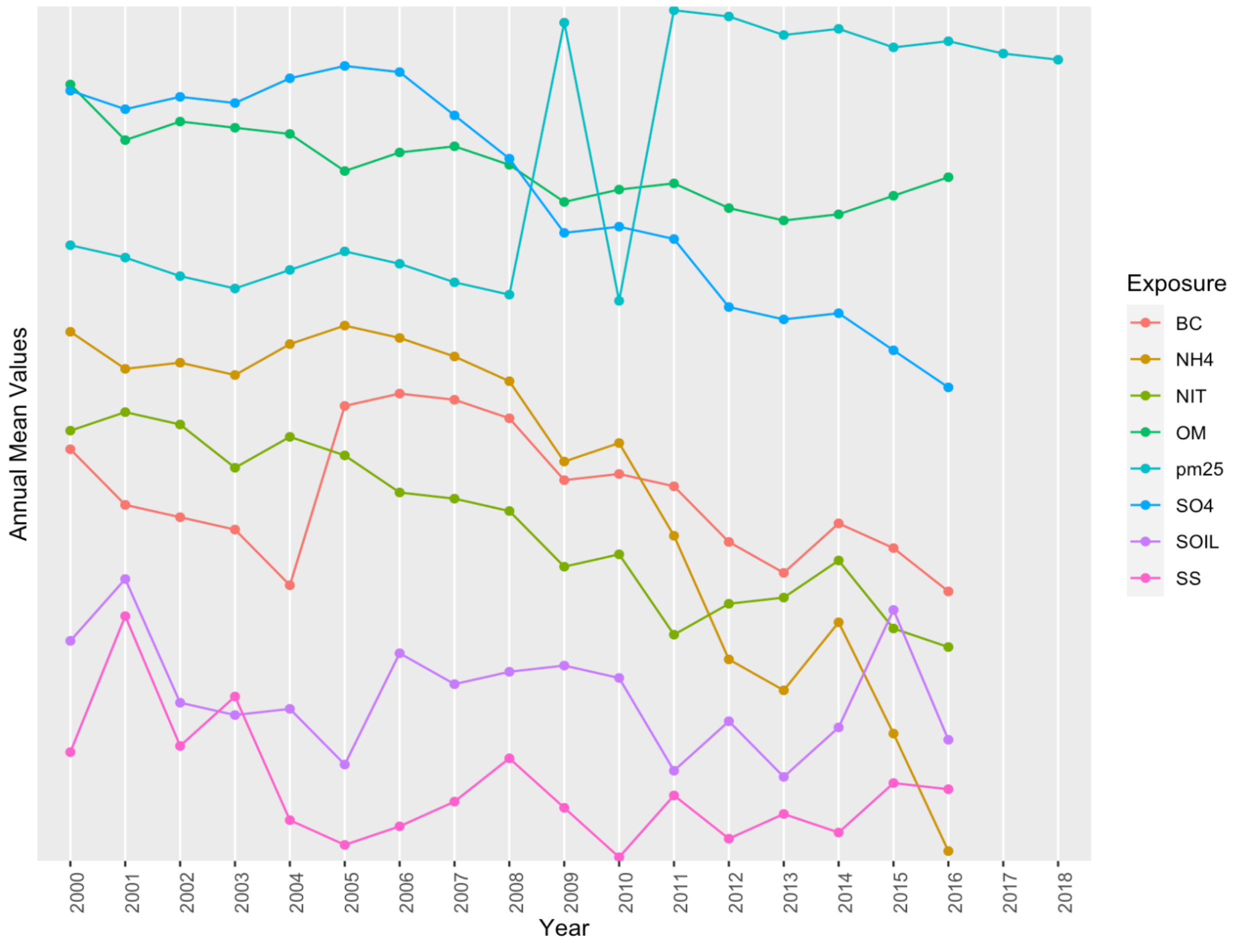

Figure S5: This figure shows the annual pollution associated with 3000 random subjects in the cohort. Again, we generally see a shift to the left over time for most of the criteria pollutants supporting that this study's use of the 15-year annual mean reflects a probable underestimate of pollutant exposure. These ridge plots also suggest that there may have been changes in data collection methodology from year-to-year in certain pollutant estimates (see organic matter (OM) and sulfur dioxide (SO_2)) and that a mean value is a better description of probable exposure.

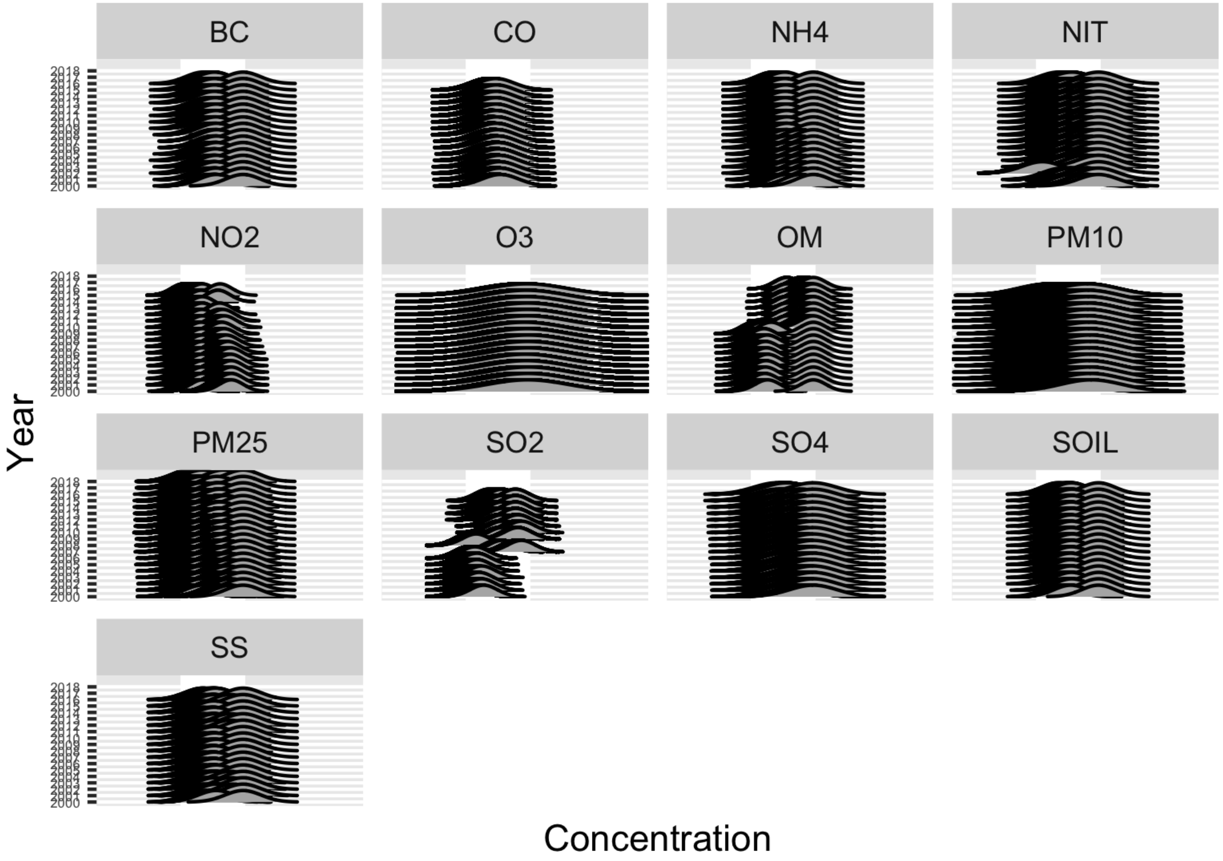

Supplement: Supplementary file 2 — Supplemental Document [file 41370_2022_502_MOESM2_ESM.pdf]
